# ARID1B, a molecular suppressor of erythropoiesis, is essential for the prevention of Monge’s disease

**DOI:** 10.1038/s12276-022-00769-1

**Published:** 2022-06-07

**Authors:** Priti Azad, Andrew B. Caldwell, Srinivasan Ramachandran, Nathanael J. Spann, Ali Akbari, Francisco C. Villafuerte, Daniela Bermudez, Helen Zhao, Orit Poulsen, Dan Zhou, Vineet Bafna, Shankar Subramaniam, Gabriel G. Haddad

**Affiliations:** 1 Division of Respiratory Medicine, Department of Pediatrics, University of California, San Diego, La Jolla, CA USA; 2 Department of Bioengineering, University of California, San Diego, La Jolla, CA USA; 3 Department of Cellular and Molecular Medicine, University of California, San Diego, La Jolla, CA USA; 4 Department of Genetics, Harvard Medical School, Boston, MA USA; 5 Broad Institute of MIT and Harvard, Cambridge, MA USA; 6 Department of Human Evolutionary Biology, Harvard University, Cambridge, MA USA; 7 Laboratorio de Fisiología del Transporte de Oxigeno/Fisiología Comparada, Facultad de Ciencias y Filosofía, Universidad Peruana Cayetano Heredia, Lima, 31 Peru; 8 Department of Computer Science and Engineering, University of California, San Diego, La Jolla, CA USA; 9 Department of Nanoengineering, University of California, San Diego, La Jolla, CA USA; 10 Department of Neurosciences, University of California, San Diego, La Jolla, CA 92093 USA; 11 Rady Children’s Hospital, San Diego, CA 92123 USA

**Keywords:** Haematological diseases, Medical genetics

## Abstract

At high altitude Andean region, hypoxia-induced excessive erythrocytosis (EE) is the defining feature of Monge’s disease or chronic mountain sickness (CMS). At the same altitude, resides a population that has developed adaptive mechanism(s) to constrain this hypoxic response (non-CMS). In this study, we utilized an in vitro induced pluripotent stem cell model system to study both populations using genomic and molecular approaches. Our whole genome analysis of the two groups identified differential SNPs between the CMS and non-CMS subjects in the *ARID1B* region. Under hypoxia, the expression levels of *ARID1B* significantly increased in the non-CMS cells but decreased in the CMS cells. At the molecular level, ARID1B knockdown (KD) in non-CMS cells increased the levels of the transcriptional regulator GATA1 by 3-fold and RBC levels by 100-fold under hypoxia. ARID1B KD in non-CMS cells led to increased proliferation and EPO sensitivity by lowering p53 levels and decreasing apoptosis through GATA1 mediation. Interestingly, under hypoxia ARID1B showed an epigenetic role, altering the chromatin states of erythroid genes. Indeed, combined Real-time PCR and ATAC-Seq results showed that ARID1B modulates the expression of *GATA1* and *p53* and chromatin accessibility at *GATA1*/*p53* target genes. We conclude that ARID1B is a novel erythroid regulator under hypoxia that controls various aspects of erythropoiesis in high-altitude dwellers.

## Red blood cell: Regulating production under low oxygen conditions

Insights into a disorder associated with excess red blood cell production in high-altitude dwellers could have broader relevance to certain cancers and blood diseases. Some people living in the Andes will eventually develop Monge’s disease, in which excessive red cell proliferation induced by low oxygen conditions leads to thickening of the blood and increased risk of heart attack or stroke. Researchers led by Gabriel Haddad of the University of California, San Diego, USA, have shown that a protein called ARID1B normally manages red blood cell levels under low oxygen conditions by regulating other genes that govern cell proliferation and survival. These same ARID1B-modulated gene networks may also contribute to various blood disorders as well as the uncontrolled growth seen in some tumor types in which cells must thrive in very oxygen-poor environments.

## Introduction

Chronic mountain sickness (CMS), or Monge’s disease, is an advancing and debilitating syndrome caused by chronic (years) exposure to high-altitude hypoxia, as experienced by people living in Cerro de Pasco in the Andes^1–9^. Excessive erythropoiesis is one of the critical traits of CMS, and this excessive pathobiological response has deleterious effects since a high hematocrit/hemoglobin increases blood viscosity and reduces blood flow to hypoxia-sensitive organs (e.g., brain and heart), often resulting in myocardial infarction or stroke in young adults^4,10^. Remarkably, there are individuals who live at that same altitude as the CMS subjects but have adapted and do not show any of the traits of the CMS individuals (non-CMS). Investigating the mechanisms underlying the difference in adaptation in the two populations provides an opportunity to learn about natural selection, genetics and the biology of adaptation in this experiment in nature.

Our whole-genome analysis of Andean high-altitude human dwellers from the two groups (CMS and non-CMS) established *ARID1B* as a key gene under selection in that population^5^. Supporting the hypothesis that ARID1B has a potential role in the regulation of erythropoiesis at high altitude, previous studies have suggested a possible role of ARID1B in erythroid differentiation^11,12^. In β-thalassemia patients, *ARID1B* is differentially expressed during erythroid development^12^, but its mechanism and role in pathology are still unknown. Mutations in *ARID1B* are associated with multiple syndromes, including Coffin-Siris syndrome, as well as several cancer subtypes, particularly acute myeloid leukemia (AML)^13,14^. ARID1B is also considered a potential therapeutic target for various carcinomas^15^. AML cells have a reduced expression of *ARID1B* compared with normal counterparts, which suggests a tumor suppressor function for ARID1B in blood^16^. Our current study is the first to identify novel regulatory functions of ARID1B in erythropoiesis under hypoxia, controlling red blood cell proliferation, apoptosis, and EPO sensitivity in subjects from the Andean region. Furthermore, our study identifies a critical role of ARID1B in regulating chromatin accessibility and gene expression of *GATA1* and *p53* in the non-CMS subjects under hypoxia.

## Materials and methods

### Patient samples and induced pluripotent stem (iPS) cell lines

All study subjects (CMS and non-CMS) were adult males residing in the Andean mountain range in Cerro de Pasco, Peru, at an elevation of ∼4338 m. CMS patients fulfilled the diagnostic criteria for CMS or Monge’s disease based on their hematocrit, O_2_ saturation, and CMS score, as described in detail in our previous studies^1,17^. Briefly, patients who manifest a chronic mountain sickness (CMS) phenotype (high hematocrit > 70, CMS score of 19–28) and non-CMS control subjects (hematocrit < 55, CMS score < 5) were included. Sea-level controls were adult males of similar age groups who lived at sea level throughout their entire life and had normal hematocrit and oxygen saturation. Each subject signed an informed written consent under protocols approved by the University of California, San Diego and the Universidad Peruana Cayetano Heredia, Lima, Peru. Deep sequencing and whole genome analysis have already been performed, and the selected DNA regions have been described in our prior publications^3,5^. We generated and characterized iPS cell lines from CMS, non-CMS and sea level subjects^1,17^. In brief, positive staining with pluripotency markers, including NANOG, Tumor-related antigen (TRA)-1-81, and alkaline phosphatase, was confirmed in these iPS cells; furthermore, RT-PCR analysis demonstrated a similar expression of prominent endogenous pluripotency genes, such as *OCT4*, *SOX2*, *c-MYC*, *KLF4*, *NANOG*, and *LIN28*, among the subjects. The differentiation potential of the three germ layers was confirmed by embryoid body (EB)-mediated differentiation in vitro. RT-PCR analysis of EBs detected a variety of differentiation markers for the three germ layers, including ectodermal markers (microtubule-associated protein 2/*MAP2* and paired box 6/*PAX6*), mesodermal markers (Msh homeobox1/*MSX1* and α-smooth muscle actin/*α-SMA*), and endodermal markers (cytokeratin 8/*CK8* and cytokeratin 18/*CK18*). The number of samples for iPSC generation was as follows: for CMS patients, *N* = 4 subjects; non-CMS subjects, *N* = 4 subjects; and sea level subjects, *N* = 2 subjects. Since we mainly focused on CMS and non-CMS individuals, we additionally tested 3 different clones for each of the 3 CMS and non-CMS subjects to assess the interclonal variability of the iPSC cell lines. Similarly, for non-CMS cells with ARID1B KD, *N* = 4 subjects, clonal variability was further tested by 3 different clones tested for each of the 3 subjects.

### RBC production using cytokines

The RBCs were generated under normoxic as well as hypoxic conditions following our previously established in vitro platform based on the protocol of Duogay’s group^1,18^.

Using this in vitro model, we thoroughly studied erythroid differentiation with appropriate CD markers and performed functional analysis of hemoglobin^1^. Briefly, for all subjects, the erythroid cultures were started with approximately 10^7^–10^8^ human iPS cells. Human iPSCs were differentiated into erythroid cells by the formation of embryoid bodies (EBs) for 27 days in a liquid culture medium with IMDM base medium (Biochrom), along with 450 µg/ml holo human transferrin (Sigma-Aldrich), 10 µm g/ml recombinant human insulin (Roche), 2 IU/ml heparin, and 5% human plasma in the presence of 100 ng/ml SCF, 100 ng/ml TPO, 100 ng/ml FLT3 ligand, 10 ng/ml rHu bone morphogenetic protein 4 (BMP4), 5 ng/ml rHu VEGF (VEGF-A165), 5 ng/ml IL-3, 5 ng/ml IL-6 (PeproTech), and 3 U/ml Epo. This process was followed by terminal differentiation as single cells with IMDM base medium (Biochrom) along with 5% human plasma, 2 IU/mL heparin, SCF (100 ng/mL), IL3 (5 ng/mL) and Epo (3 IU/mL). For all erythroid assays, *N* = 4 subjects for each of the CMS and non-CMS samples. Additionally, we tested multiple clones (3 clones) for each of the CMS, non-CMS and non-CMS (with ARID1B KD) samples to show interclonal differences between erythroid cultures prepared from each iPS cell line. We measured the hematopoietic potential of these iPSCs, and details regarding their basic characteristics are shown in Supplementary Fig. 3 and Supplementary Table 1.

### Hypoxia regimen and FACS analysis

The erythroid cells at the EB stage were cultured for 1 week at 37 °C in 5% CO_2_/air. After 1 week, these cells were transferred to a hypoxic incubator set at 37 °C, 5% O_2_, and 5% CO_2_ for 3 weeks. Subsequently, the cells underwent FACS analysis (FACSCanto cell analyzer (BD) using FACSDiva software, version 6.0; BD), and Glycophorin A (BD Biosciences, cat. 340947) was used as a marker for the assessment of RBC production and maturation. For FACS analysis, erythroid cells were analyzed for *N* = 4 subjects for CMS, non-CMS and non-CMS with ARID1B-KD samples. Additionally, we tested multiple clones (3 clones) for each of the subjects to show interclonal differences between erythroid cultures prepared from each iPS cell line.

### BFU-e and CFU-e assays

FACS-sorted iPS-derived CD34 + cells from the CMS and non-CMS subjects were plated at a density of 10^5^ cells per 35 mm dish combined with MethoCult Optimum media (Stemcell Technology, Cat. H4034) and 2% FBS (Sigma, Cat. F2442). Dishes were incubated at 37 °C in an incubator with 5% CO_2_ and 5% O_2_ for 14 d, at which time colonies were scored for BFU-E and CFU–GEMM (granulocyte, erythrocyte, monocyte, megakaryocyte). For EPO dose-response experiments, Methocult classic (without EPO) (Stemcell Technology, Cat. H4434) was mixed with various doses of EPO (0.5, 1, 1.5, 2, 2.5, 3 and 3.5 units) and assayed for colony production. We used *N* = 4 subjects for each group that was tested by this assay.

### ROS measurement and antioxidant treatment

We used the 2,7 dichlorofluorescein diacetate method of ROS measurement described by Lee et al.^19^. Erythroid cells at the EB stage were homogenized with 500 μl of PBST (PBS containing 0.1% Tween 20), and 100 μl of each supernatant was transferred to a 96-well plate. Then, 2, 7 dichlorofluorescein (Invitrogen, Cat. D399) was added to each sample at a final concentration of 50 μm, and the fluorescence intensity was measured at excitation of 485 nm and emission of 640 nm and quantified using a fluorescence microplate reader (Biotek, Synergy 2, VT). The fluorescence intensities were normalized to the protein levels. Each experiment was performed with three replicates. The oxidation-insensitive counterpart of dichlorofluorescein dye (C369, Invitrogen) was used as a control to ensure that the changes in the fluorescence observed with the oxidation-sensitive dye DCFH were solely due to changes in ROS production. For the antioxidant treatment, N-acetyl-L-cysteine (Sigma, Cat. A9165) was added to the culture medium at 1 mM twice a week. *N* = 4 subjects for the non-CMS and non-CMS with ARID1B-KD groups.

### Apoptosis assays

Apoptosis analysis was performed using an APC Annexin V Apoptosis Detection Kit with PI (Biolegend, Cat. 640932). The analysis was performed based on the manufacturer’s instructions. Briefly, the cells were washed twice with cold BioLegend’s Cell Staining Buffer and then resuspended in Annexin V Binding Buffer at a concentration of 0.5 × 10 cells/ml. We transferred 100 µl of cell suspension and added 5 µl of APC Annexin V and propidium iodide solution. The cells were vortexed gently and incubated for 15 min at room temperature (25 °C) in the dark. Finally, 400 µl of Annexin V Binding Buffer was added to each tube and analyzed by flow cytometry using a FACSAria II System (BD Biosciences). Apoptosis assays were conducted on human iPSC-derived erythroid cultures after 2 weeks of exposure to hypoxia. For each group, *N* = 4 subjects.

### Real-time PCR analysis for measurement of gene expression

Erythroid cells at the EB stage were used after 1, 2, or 3 weeks in culture under hypoxia. RNA was isolated from EBs using the RNeasy Mini kit (Qiagen, Cat. 74104). cDNA was produced from total RNA through RT-PCR using a Superscript Vilo IV system (Invitrogen, Cat. 11756050). Real-time PCR was performed using a GeneAmp 7900 sequence detection system using POWER SYBR Green chemistry (Thermo Fisher, Cat. 4368577). The primer sequences used were as follows:

ARID1B-L-, 5’-CAGCAGATGCCACCTCAGTA3’; ARID1B-R-5’-GCTGCTGGCTGTAATATGGC-3’; GATA1-L, 5′-CCTGCTTTGTTGCCAATG-3′; GATA1-R, 5′-CTGCTCCACTGTTACGGATAC-3′; p53-L-, 5’-GCCCAACAACACCAGCTCCT-3’; p53-R-, 5’-CCTGGGCATCCTTGAGTTCC-3’. The expression level of GAPDH was used to normalize the results. GADPH-L, 5′-CTGGCATTGCCCTCAACGACC-3′ and GADPH-R, 5′-CTTGCTGGGGCTGGTGGTCC-3′

For Real-time PCR analysis, 4 subjects were tested in each group.

### Lentiviral vectors and transduction of iPSCs for the generation of knockdown and overexpression cell lines

The human ARID1B (shRNA) lentiviral system construct was purchased from GE Healthcare (Dharmacon, Cat. VGH5518-200172117). p-53 shRNA was purchased from Vector Builder (Cat. VB180711-1087xsr). The packaging and lentivirus generation were performed by the Salk Institute Gene Transfer, Targeting, and Therapeutics Core. Transduced cells were selected with 0.5 µg/ml puromycin (Sigma-Aldrich, Cat. P8833) or blasticidin (EMD Millipore, Cat. CAS 589205). For double KD, puromycin and blasticidin combinations were used for selection. The expression of each construct was verified by real-time PCR. For each gene manipulation, 4 subjects were tested and used for further analysis in each group.

### ATAC-sequencing, data processing, and enrichment analysis

ATAC-Seq transposition experiments were performed on 50,000 non-CMS and non-CMS with ARID1B KD (replicates, *n* = 3 subjects) hiPSC-derived erythroid cells grown under hypoxic and normoxic conditions for two weeks. The cells were washed with PBS followed by lysis with cold lysis buffer (10 mM Tris-HCl, pH 7.4, 10 mM NaCl, 3 mM MgCl2, 0.1% IGEPAL CA-630). The cells were then suspended in 50 μl of 1X reaction buffer (25 μl of Tagment DNA Buffer, 2.5 μl of Tagment DNA enzyme I, and 22.5 μl of water) (Illumina, Cat. 15028523) as previously described by Buenrostro et al. 2013^20^. Transposase reactions were carried out at 37 °C for 30 min, and DNA was purified using ChIP DNA Clean & Concentrator kits (Zymo Research, Cat. D5205). DNA was amplified using dual-barcoded primers (Illumina) and NEBNext High-Fidelity 2XPCR Master Mix (NEB, Cat. M0541S) for 14 cycles. The resulting libraries were sequenced on an Illumina Novaseq platform generating paired-end, 50 bp (PE50) reads with an average of over 100 million reads per sample.

ATAC-Seq data preprocessing was performed with TrimGalore!, removing sequencing adaptors and selecting all paired-end reads above a quality score threshold (Phred Q > 20). Trimmed reads were aligned to the hg19 human genome (1000 Genomes reference) using BBMap v37.95 in the BBtools suite with the options maxindel = 20 ambig = random, followed by sorting and indexing of bam files using Samtools v1.35 and annotation of PCR duplicates using the Picard v2.3.0 markDuplicates function with the option VALIDATION_STRINGENCY = LENIENT. All duplicates and mitochondrial, chromosome X, chromosome Y, and EBV reads were removed using Samtools v1.3 command view with the options -b -h -f 3 -F 4 -F 8 -F 256 -F 1024 -F 2048. Genrich v0.6 was used to call peaks on the ATAC-Seq data to determine regions of accessible chromatin, using all three replicates to generate a consensus list of peaks for each condition. The R package *Diffbind* v2.8.0^21^ was used to identify peaks with differential accessibility between the non-CMS and non-CMS ARID1B KD conditions with CONDITION_CONSENSUS peak selection and *edgeR* for differential analysis. Differentially accessible chromatin peaks were defined as those passing a threshold of |FC | > 1.5 and FDR *P* < 0.05 occurring in a promoter or enhancer region. The differential peaks were annotated using the R package ChIPseeker^22^, defining the promoter region -1000 to 500 bp from the TSS. Enhancer-associated ATAC-Seq regions were defined as differential peaks occurring within the region list of TSS-associated enhancers generated by the FANTOM5 project using the join_overlap_inner function in the plyranges R package^23^. De novo motif generation for ARID1B was performed with ARID1B ChIP-Seq in K562 cells generated by the ENCODE project with GimmeMotifs^24^ using the Homer^25^ algorithm. Motif enrichment of the three ARID1B-associated de novo motifs was performed similarly to the option-known. To identify candidate transcriptional regulators potentially associated with ARID1B, we performed enrichment analysis of differential ATAC-Seq regions using the logistic regression model test function chipenrich in the *chipenrich* R package^26^. The locus definition nearest_tss was used for enrichment of promoter-located peaks, whereas the 1 kb_outside definition was used for enhancer-located peaks with the ENCODE-ChEA consensus TF-gene target database^27–29^. Promoter and TSS-associated enhancer heatmaps were generated with the deepTools functions *computeMatrix* and *plotHeatmap*. We carried out HINT-ATAC^30^ differential footprinting motif activity analysis between the ARID1B KD and non-CMS under hypoxia groups. For this, the HOCOMOCOv11^31^ and CIS-BP^32^ motif databases were used for motif matching for the footprints found in the enhancer regions (*P*-value < 0.01). For the ATAC-Seq TF regulatory network, we used the union of TF-gene edges from the ENCODE-ChEA consensus database and the ReMap database^33^ and projected the differential z score of significant motifs from HINT-ATAC analysis for TFs targeted by GATA1, GATA2, or p53 regulators.

## Results

### ARID1B prevents hypoxia-induced excessive erythropoiesis (EE) in Monge’s disease

**Fig. 1:**
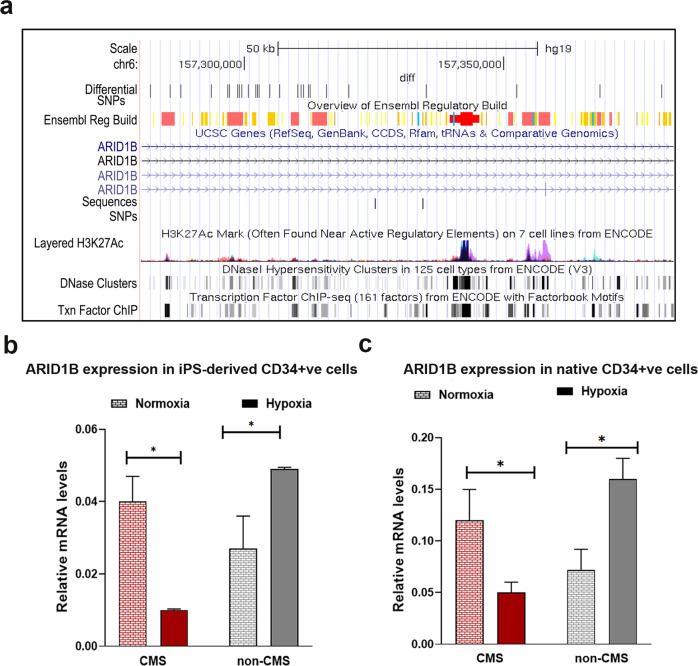
*ARID1B* is differentially regulated and expressed in CMS and non-CMS subjects and cells under hypoxia. **a** The fig. shows differentially expressed SNPs in the *ARID1B* region. There were 26 SNPs that were significantly different in frequency in the CMS and non-CMS subjects. The SNPs overlap with transcriptional regulators (shown in red), enhancers (orange), and transcriptional binding sites (gold). SNPs overlapping with DNase I hypersensitive sites as well as H3K27Ac (found near active regulatory elements) as determined by a ChIP-seq assay (ENCODE project) are also shown in the fig. **b** *ARID1B* is differentially expressed in the CMS and non-CMS cells under hypoxia (both iPS-derived cells), as determined by qPCR. The fig. shows the relative mRNA levels under normoxia (pattern) and hypoxia (5% O_2_) (fill). *P < 0.05. Each bar represents the mean, and error bars represent the SE with *N* = 4 iPS-derived cells. For each sample, the levels were measured in triplicate. Each experiment was repeated three times. **c** *ARID1B* is differentially expressed in the CMS and non-CMS cells under hypoxia (native CD34 + ve) as determined by qPCR. The fig. shows the relative mRNA levels under normoxia (pattern) and hypoxia (5% O_2_) (fill). *P < 0.05. Each bar represents the mean, and error bars represent the SE with *N* = 5 subjects cells for native CD34 + . For each sample, the levels were measured in triplicate. Each experiment was repeated three times.

**Fig. 2:**
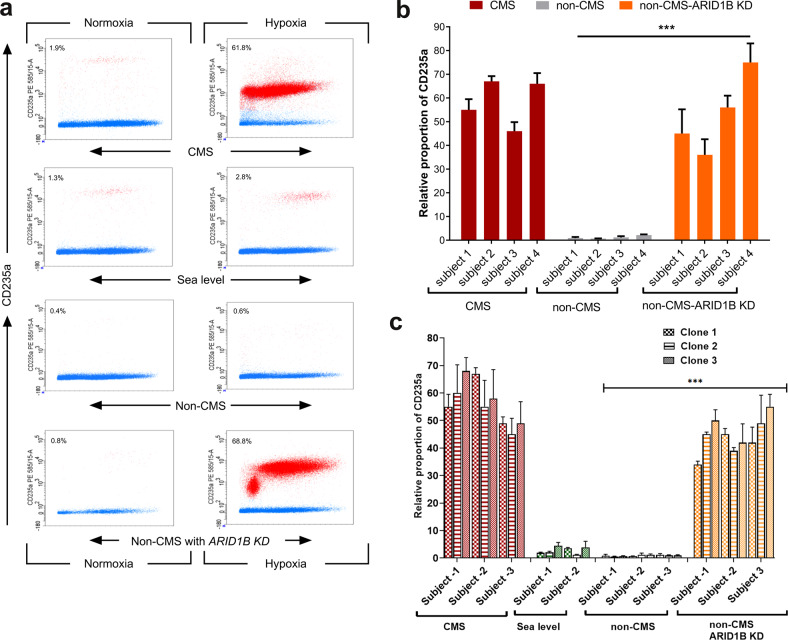
ARID1B is a critical regulator of excessive RBC production in non-CMS subjects under hypoxia. **a** KD of ARID1B in non-CMS cells increases the proportion of CD235a only under hypoxia. FACS plots represent the proportion of CD235a and PI in the CMS (top), sea level (middle) and non-CMS (bottom) cells under normoxia and hypoxia. The bottommost panel FACS plot shows the ARID1B KD phenotype in non-CMS cells. The fig. shows marked differences in the non-CMS subjects under hypoxia with the KD of ARID1B under hypoxia. In the FACS plots, the cells in red represent CD235a + cells, and the blue dots represent PI + cells. FACS analysis was gated based on size and the CD235a marker. In the FACS plot for all the CMS, non-CMS, and non-CMS samples with ARID1B, we observed a population of cells representing a scatter plot of CD235a + cells (red dots) and PI + cells (blue dots). The proportion of CD235a is calculated based on live CD235a + cells and is shown on the top left of the plot (e.g., 68.8% for non-CMS-ARID1B under hypoxia). **b** Multiple subjects were tested to study the effect of ARID1B on RBC production. The fig. shows the summary of ARID1B KD under hypoxia in multiple subjects. *N* = 4 subjects for the CMS, non-CMS and non-CMS cells with KD of ARID1B. Each bar represents the mean, and error bars represent the SE with *N* = 4. For each sample, the levels were measured in triplicate. Each experiment was repeated three times. ***P < 0.001 (t-test). **c** Multiple clones were studied for each subject. The Fig. depicts ARID1B KD under hypoxia in multiple clones in multiple subjects. Three subjects were tested in each group: CMS, non-CMS and non-CMS with ARID1B KD. Three clones were tested for each subject to assess interclonal variability. Each bar represents the mean, and error bars represent the SE with *N* = 3. For each sample, the levels were measured in triplicate. Each experiment was repeated three times. ***P < 0.001 (*t*-test).

Our whole genome analyses performed on subjects with Monge’s disease identified genome-wide regions with significant differences in haplotype frequencies consistent with selective sweeps^5^. One of the selected regions included the *ARID1B* gene^5^, with 26 SNPs identified as significantly divergent in allele frequency between CMS and non-CMS individuals (differential SNPs) (Fig. 1a). These SNPs overlap with transcriptional regulators, enhancers, and transcriptional binding sites of factors that are involved in erythropoiesis, such as RUNX3, STAT3, STAT5, and GATA1. Although ARID1B has been linked to physiologic and pathophysiologic red blood cell fate, its role in erythropoiesis is not well defined, especially in regard to Monge’s disease. Since ARID1B is located in a high-altitude selected genomic region, we asked if it is involved in the regulation of RBC levels in high-altitude dwellers. We first examined whether *ARID1B* is differentially expressed in CMS and non-CMS individuals under hypoxia. Figure 1b shows that *ARID1B* expression was significantly downregulated in the CMS subjects but upregulated in the non-CMS subjects during hypoxia in iPS cells. We further strengthened our hypothesis of a critical role of ARID1B under hypoxia in this population by validating our results using native CD34 + cells (Fig. 1b). Indeed, we found significant expression differences in *ARID1B* in the CMS and non-CMS native CD34 + cells under hypoxia (Fig. 1c). To elucidate the role of ARID1B in the non-CMS subjects, we knocked down its expression using lentivirus shRNA in subject-derived iPS cells. We then selected clones that showed significant knockdown (KD) (>90%) of ARID1B expression, as determined by real-time PCR (Supplementary Fig. 1). Strikingly, ARID1B KD in the non-CMS cells had a substantial impact on erythroid cell levels: it converted the non-CMS phenotype into a CMS phenotype in terms of excessive erythropoiesis. Indeed, the proportion of erythroid cells increased remarkably in hypoxia, as measured by CD235a or Glycophorin A (Fig. 2a). The non-CMS cells under hypoxia (5% O_2_ for 3 weeks) had a small relative proportion of CD235a (0.6%) in the wild type, which increased significantly (68.8%) with ARID1B KD (*P* < 0.0001). This is a remarkable100-fold increase in RBCs when *ARID1B* expression was downregulated. This result was consistent in multiple subjects as well as multiple clones from each subject (3 clones per subject and at least 3 subjects per group) (Fig. 2b, c). Hence, *ARID1B* expression under hypoxia seems to constrain or limit RBC levels in non-CMS subjects.

### ARID1B affects high altitude-induced erythropoiesis through GATA1 via ROS mediation

**Fig. 3:**
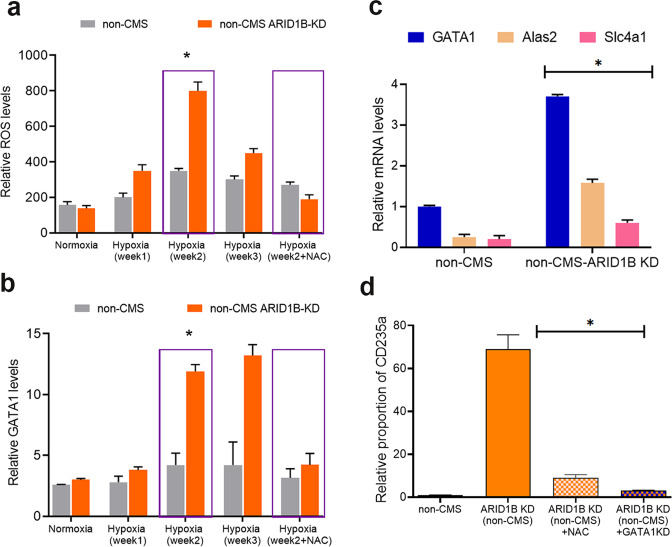
ARID1B KD changes *GATA1* expression and RBC production through ROS mediation. **a** KD of ARID1B increased ROS levels: non-CMS (gray) and non-CMS with KD-ARID1B (orange). KD of ARID1B increased ROS levels under hypoxia. ROS levels were the highest at week 2, as shown by the purple box. The last column shows the effect of the addition of the antioxidant NAC at week 2. *P < 0.05 (*t*-test). Each bar represents the mean, and error bars represent SE with *N* = 4. Each experiment was repeated three times. **b** The fig. shows the relative mRNA levels of *GATA1* under hypoxia in the non-CMS (gray) and non-CMS with KD-ARID1B groups (orange). As shown in the fig., the increase in GATA1 is the most in week 2, coinciding with the increase in ROS. Each bar represents the mean, and error bars represent the SE with *N* = 4. Each experiment was repeated three times. **c** Real-time PCR results of the ARID1B KD and non-CMS subjects. ARID1B KD significantly increased the *GATA1* levels as well *GATA1* target genes *Alas2* and *Slc4a1*. *P < 0.05. Each bar represents the mean, and error bars represent the SE with *N* = 4. Each experiment was repeated three times. **d** The fig. shows the production of erythroid cells (as relative proportion of CD235a) in the non-CMS and non-CMS with KD-ARID1B groups. As shown in the fig., the addition of the antioxidant NAC or KD of GATA1 leads to a significant reduction in RBC production. *P < 0.01 (*t*-test). Each bar represents the mean, and error bars represent the SE with *N* = 4. Each experiment was repeated three times.

Importantly, the exaggerated erythropoietic response was present predominantly under hypoxia (5% O_2_) when we downregulated the expression of *ARID1B*. This led us to query if hypoxia mediators, such as ROS, could have an impact on the erythropoietic response in these erythroid cells. In fact, some studies have suggested that ROS can enhance erythropoiesis^34^. We therefore hypothesized that ARID1B overexpression decreases ROS and that ARID1B KD increases ROS production. As shown in Fig. 3a, ROS levels were significantly increased with ARID1B KD in the non-CMS cells, with a peak after two weeks of hypoxia. This begged the question as to whether the ROS increase with ARID1B KD led to enhanced erythropoiesis by modulating downstream factors in non-CMS subjects.

To further explore the molecular mechanisms of ARID1B in erythropoiesis, we measured the expression levels of erythropoietic transcription factors and their downstream effectors in erythroid cells after exposure to hypoxia and normoxia as described in the Methods. We did not observe major differences in the relative expression levels of *STAT5*, *STAT3*, and *EPOR* in the CMS and non-CMS subjects under hypoxia. However, we observed significant differences in GATA1 between the 2 populations (Fig. 3b). With ARID1B KD, *GATA1* increased significantly in the non-CMS group during hypoxia (3-fold change, *P*-value = 0.001) (Fig. 3b). Interestingly, the increase in GATA1 coincided with the increase in ROS production at week 2 (Fig. 3a, b). We therefore tested whether ARID1B prevents erythropoiesis by modulating GATA1 levels through ROS. In fact, the addition of the antioxidant N-acetyl-L-cysteine (NAC) largely abolished the upregulation of GATA1 expression (Fig. 3b). Furthermore, the increase in *GATA1* expression affected downstream *GATA1* target genes, red cell maturation and survival of erythroid cells. Indeed, the increase in *GATA1* expression enhanced the expression of *GATA1* target genes such as *ALAS2* and *Slc4a1* (Fig. 3c), indicating that GATA1 signaling was activated. Our results therefore confirm that addition of NAC as well as KD of GATA1 controlled the excessive RBC phenotype in ARID1B KD in non-CMS subjects (Fig. 3d). This finding strongly implies that ARID1B modulates GATA1 levels by regulating ROS in non-CMS subjects. Since a) GATA1 affects various stages of erythropoiesis and b) erythropoiesis involves both proliferation and apoptosis, we asked if ARID1B KD changes the proliferative capacity or apoptotic rate in the non-CMS subjects at specific stages of erythropoiesis. We therefore performed proliferative assays, such as BFU and CFU assays, as well as apoptosis measurements at various erythroid stages.

### ARID1B KD leads to increased proliferation and EPO sensitivity through the regulation of p53 levels

**Fig. 4:**
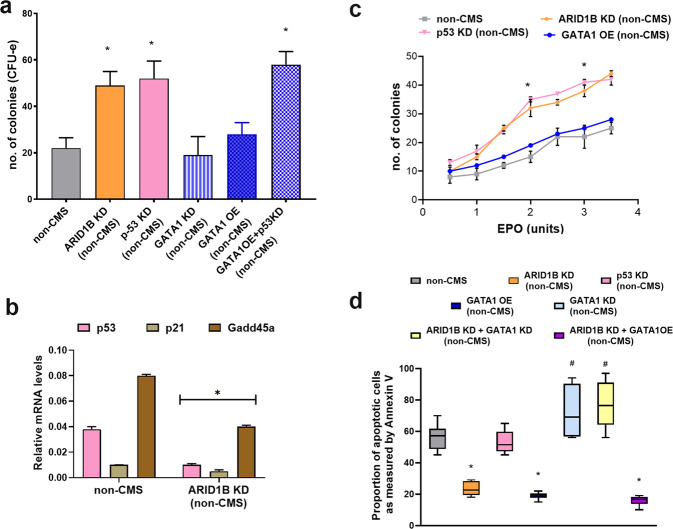
ARID1B regulates proliferation and EPO sensitivity (via p53) and apoptosis (through GATA1) in erythroid cells. **a** The graphs depict CFUs under hypoxia in the following genotypes: (a) non-CMS, (b) non-CMS (ARID1B KD), (d) non-CMS (p53 KD), (e) non-CMS (GATA1 KD), (f) non-CMS (GATA1 OE) and (g) non-CMS (GATA1OE + p53 KD). The KD of p53 plays a major role in the proliferation of CFU units, suggesting that ARID1B KD controls proliferation by p53 KD. *P < 0.05 (*t*-test). Each bar represents the mean, and error bars represent the SE with *N* = 4. **b** Real-time PCR results of the ARID1B KD and non-CMS subjects. ARID1B KD significantly decreases p53 and its target genes (*p21* and *Gadd45a*). *P < 0.05 (*t*-test). Each bar represents the mean, and error bars represent the SE with *N* = 4. Each experiment was repeated three times. **c** The Fig. shows the effect of EPO sensitivity (as measured by EPO dose response and colony forming potential) with ARID1B KD. By performing EPO dose response experiments, we observed that ARID1B changes EPO sensitivity through the KD of p53 levels. Each point represents the mean, and error bars represent the SE with *N* = 4. *P < 0.05 (*t*-test). **d** Apoptosis under hypoxia as measured by FACS using Annexin V: The graph depicts apoptosis under hypoxia at the EB stage after 2.5 weeks. The graphs show various combinations of knockdown or overexpression of GATA1, p53 and ARID1B. i) Non-CMS, ii) ARID1B KD (non-CMS), iii) p53 KD (non-CMS) iv) GATA1 OE (non-CMS), v) GATA1 KD (non-CMS), vi) ARID1B KD + GATA1 KD (non-CMS), vii) ARID1B KD + GATA1 OE (non-CMS). An increase in GATA1 by ARID1B (KD) leads to decreased apoptosis in non-CMS subjects and thereby leads to increased RBC production under hypoxia. The number of subjects in each group is *N* = 4, and the horizontal line within each box denotes the median. *P < 0.05, a significant decrease in apoptosis (*t*-test). #*P* < 0.05, a significant increase in apoptosis (*t*-test).

Our colony-forming assays showed that ARID1B KD leads to increased proliferative potential, specifically at the CFU-e stage, with the number of colonies doubling in the non-CMS cells (Fig. 4a). Since *GATA1* inhibits p53^35^ and OE of ARID1B has been shown to stabilize p53 levels and affect cellular proliferation^36^, we hypothesized that *ARID1B* regulates p53 levels in these non-CMS subjects. Interestingly, real-time PCR analysis showed that ARID1B KD led to significant downregulation of the expression of *p53* and its target genes (*p21* and *Gadd45a*) (Fig. 4b). To further evaluate whether the expression changes of *p53* or *GATA1* have a functional role in proliferation, we separately knocked down p53 and GATA1 and individually studied their effects on the colony forming potential. GATA1 KD or overexpression (OE) had a modest influence on colony production. For example, GATA1 KD resulted in a slight and insignificant change in CFU-e levels in the non-CMS cells (19 CFUs for GATA1 KD vs. 22 CFUs for non-CMS). Similarly, GATA1 OE increased the colony number to 28 CFU-s compared to 20 CFU-s for the controls (*P* = 0.051, *t*-test). However, p53 KD led to a 3-fold increase in CFU-e production in the non-CMS cells (*P* < 0.001, Fig. 4a). These results indicate that ARID1B controls proliferation through p53 at the CFU erythroid stage in non-CMS subjects. Our results thus demonstrate a modest increase in CFU-e in the GATA1 OE cells but a remarkable increase in CFU-e in the p53 KD cells.

Since the CFU-e stage of erythropoiesis is very EPO-sensitive and a previous study suggested that the expression of ARID1B coincided temporally with that of EPO expression^12^, we asked whether ARID1B affects EPO sensitivity. Figure 4c shows that ARID1B KD in the non-CMS cells significantly increased EPO responsiveness as the number of colonies increased 3- to 4-fold (Fig. 4c). Our expression studies showed that ARID1B KD results in upregulation of GATA1 expression (Fig. 3c) and downregulation of p53 expression (Fig. 4b), which led us to investigate whether these expression changes are associated with EPO sensitivity. Indeed, p53 KD has a similar response as ARID1B KD (Fig. 4c). We found that with p53 KD, the EPO responsiveness of the non-CMS subjects increased significantly. At the same level of EPO, the non-CMS cells doubled their colony production (15 colonies vs. 30 colonies) with p53 KD (*p* < 0.001, *t* test), demonstrating that ARID1B prevents the increase in RBC levels through p53 under hypoxic conditions. In addition to proliferation, we next asked whether ARID1B controls RBC levels by changing the apoptotic rates of erythroid cells.

### ARID1B maintains apoptosis rates in erythroid cells in the Andean population through GATA1

When we knocked down ARID1B in non-CMS cells, the apoptosis rates were significantly decreased (23%; *P* < 0.01) (Fig. 4d). To prove that ARID1B KD decreases apoptosis and leads to better survival through GATA1, we first designed approaches involving single and double KD of ARID1B and GATA1. For example, when we knocked down ARID1B alone in non-CMS cells, apoptosis dropped by more than half (from 56% to 23%) (Fig. 4d). KD of p53 alone resulted in an apoptosis rate similar to that of wild-type non-CMS cells. Single KD of GATA1 substantially increased apoptosis, and OE of GATA1 decreased apoptosis levels in the non-CMS cells. However, the double KD of ARID1B and GATA1 in non-CMS cells increased apoptosis to 74%, which strongly suggests that GATA1 is the key downstream mediator of apoptosis. Second, with ARID1B KD and GATA1 OE in the non-CMS cells, apoptosis rates were comparable to ARID1B KD (Fig. 4d). Therefore, these experiments demonstrate that ARID1B regulates apoptosis in the non-CMS cells through GATA1. This finding is consistent with previous studies that have observed a critical role of GATA1 in regulating apoptosis in erythroid cells^37^. Notably, ARID1B seems to regulate both facets of erythropoiesis, proliferation (through p53) and apoptosis (via GATA1), thereby highlighting its essential role in controlling hypoxia-induced excessive erythropoiesis.

### ARID1B modulates chromatin accessibility at *GATA1* and *p53* target genes

**Fig. 5:**
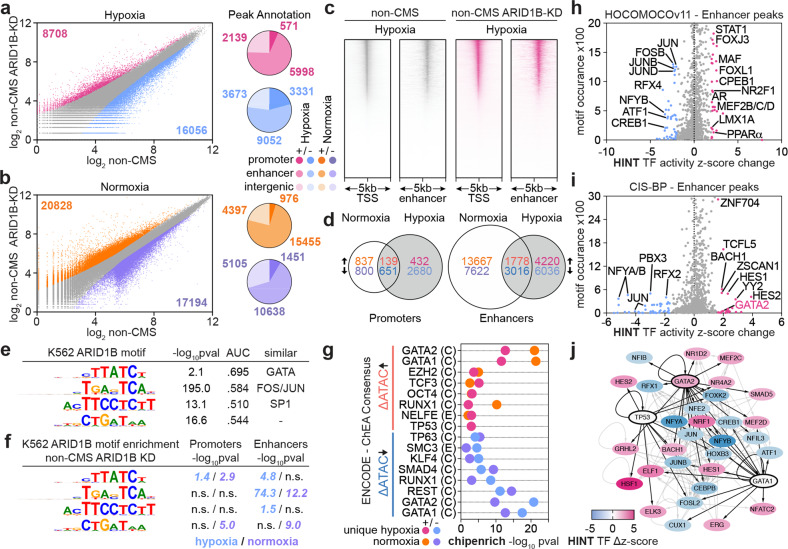
ATAC-seq identifies differential regulation of GATA1 and p53 targets by ARID1B in erythroid cells. **a**, **b** Differential ATAC-Seq peaks in the non-CMS ARID1B KD cells relative to the non-CMS cells under (**a**) hypoxic or (**b**) normoxic conditions; left, log_2_ expression of all ATAC-Seq peaks (red, significantly increased accessibility; blue, significantly decreased accessibility; adj. *p*-value < 0.05); right, proportion of differential ATAC-Seq peaks occurring in promoter regions, TSS-associated enhancer regions, or intergenic regions. **c** ATAC-Seq read coverage plot of chromatin accessibility occurring within 5 kb of promoter (TSS) or enhancer regions in the non-CMS and non-CMS ARID1B KD under hypoxia groups. **d** Overlap of differentially accessible ATAC-Seq peaks in the non-CMS ARID1B KD samples occurring within promoters or enhancers between normoxic and hypoxic conditions. **e** GimmeMotifs de novo motif generation of ENCODE ARID1B ChIP-Seq in K562 cells; -log_10_pval of motif enrichment, ROC AUC of each motif, and TF motif type best matching each de novo motif. **f** GimmeMotifs motif enrichment of de novo ENCODE K562 ARID1B ChIP-Seq motifs from E in decreasing differentially accessible ATAC-Seq peaks occurring within promoters or enhancers in the non-CMS ARID1B KD cells under hypoxia and normoxia. **g** Chipenrich geneset peak enrichment of differential enhancer ATAC-Seq peaks either increasing (top, pink) or decreasing (bottom, blue) in the non-CMS ARID1B KD cells under hypoxia or normoxia using ENCODE-ChEA. Consensus TF-target geneset database;. (E) = ENCODE, (C) = ChEA. **h**, **i** HINT TF footprinting analysis in differentially accessible ATAC-seq enhancer regions in the ARID1B KD cells under hypoxia using the (**h**) HOCOMOCOv11 or (**i**) CIS-BP motif database to identify TFs with a change in footprinting activity. Red, significantly increased activity; blue, significantly decreased activity (*p*-value < 0.05). **j** Regulatory network of differentially active TFs by HINT-ATAC analysis based on the ENCODE-ChEA Consensus and ReMap TF-gene databases with GATA1, GATA2 and TP53 (p53). Black arrows represent regulatory TF-gene edges from GATA1, GATA2, or p53 to significant HINT-ATAC TFs. Gray arrows represent regulatory TF-gene edges between all other TFs significantly identified by HINT-ATAC. Color mapping (red-blue) represents the HINT-ATAC differential z score.

ARID1B is a subunit of the SWI/SNF complex, which mediates modifications of the chromatin structure that are critical in modulation of gene expression of various transcriptional regulators involved in hematopoiesis. Indeed, SWI/SNF has been shown to be directly involved in regulating chromatin accessibility at transcription start sites (TSSs) and enhancers of target genes of various hematopoietic transcriptional regulators, such as EPO, HIF, FOS/JUN (AP1) and the GATA family^38^, under low O_2_ conditions. Interestingly, impairment of the SWI/SNF complex renders cells resistant to hypoxia-induced cell cycle arrest. Previous work has demonstrated that KD of ARID1B leads to widespread modification of the chromatin landscape, particularly at enhancer regions^39^. Since ARID1B is important in hypoxia, is a subunit of the SWI/SNF chromatin modifying complex, and regulates GATA1 expression, we next sought to determine if it regulates erythropoiesis by modifying the chromatin state and accessibility of transcriptional regulators such as GATA1 and p53. This question stems from our observations that GATA1 and p53 have a functional role in modulating the proliferation and apoptosis of developing red blood cells. We thus performed ATAC-Seq in the non-CMS and non-CMS ARID1B KD cells under normoxic and hypoxic conditions. This experiment revealed a substantial number of differentially accessible chromatin peaks in the non-CMS ARID1B KD cells compared to the non-CMS wild-type cells (without an ARID1B KD), with the majority of peaks occurring within TSS-associated enhancer regions under both hypoxia and normoxia (Fig. 5a–d). De novo motif generation for ARID1B, using a previous ARID1B ENCODE ChIP-Seq study in K562 cells, identified four significant TF motifs, three of which had similarity to known erythroid TFs: GATA family members (GATA1/2/6), FOS/JUN (AP1), and SP1 (Fig. 5e). The motif enrichment analysis using these de novo ARID1B motifs in the promoter and enhancer regions showed significant enrichment of the GATA and the FOS/JUN (AP1) motifs in the ARID1B KD cells under hypoxia (Fig. 5f). As the *p53* (*TP53*) target gene *p21* (CDKN1A) showed downregulated expression upon ARID1B KD under hypoxia, we investigated the chromatin accessibility profile of this gene, revealing decreased accessibility at the promoter regions for *CDKN1A* (-1.60 log2FC, FDR P 6.11 × 10-5), in alignment with the decreased gene expression observed by qPCR (Fig. 4b and Supplementary Fig. 2). We next sought to identify the TFs whose downstream target genes were enriched for differential accessibility within enhancer regions using Chipenrich with the ENCODE-ChEA Consensus TF-target gene database. Interestingly, *GATA1* and *GATA2* target genes were the top significantly enriched genes for differential chromatin accessibility in enhancers for the ARID1B KD cells under both hypoxia and normoxia (Fig. 5g). Furthermore, *p53* target genes were significantly enriched within enhancer regions with increasing accessibility in the ARID1B KD hypoxia condition. To identify transcription factors with differential activity upon ARID1B KD under hypoxia, we performed HINT footprinting analysis in the ARID1B KD differentially accessible enhancer regions. In the HOCOMOCOv11 motif database, HINT identified increased activity of TFs associated with erythropoiesis and some of those being activated by GATA1, including MEF2C^40^, PPAR^41,42^, and LMX1A^43,44^ (Fig. 5h). Similar activation of erythropoiesis regulators was observed using the CIS-BP motif database, with GATA2, YY2, and BACH1 demonstrating increased activity (Fig. 5i). A substantial number of these TFs with significant differential footprinting activity were regulated by GATA1, GATA2, or p53 in the ENCODE-ChEA Consensus and ReMap TF-target databases, as shown in Fig. 5j. These results also demonstrate that, in line with previous studies, ARID1B KD results in a decrease in chromatin accessibility at enhancers primed for FOS/JUN binding. Moreover, ARID1B KD results in significant chromatin modification at the TSS-associated enhancer regions of *GATA1*, *GATA2*, and *p53* target genes.

**Fig. 6:**
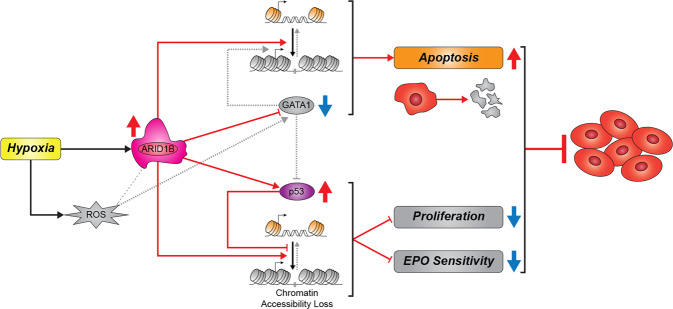
Schematic diagram showing adaptive mechanisms at high altitude: The critical role of ARID1B in preventing Monge’s disease. Based on the experiments comparing adapted non-CMS cells vs. non-CMS with ARID1B-KD cells, we can summarize the adaptive advantage of the non-CMS cells using the model shown above. Under hypoxia, ARID1B is upregulated in the non-CMS adapted population. Increased expression of ARID1B in the non-CMS cells helps in regulating RBCs levels under hypoxia by. i) keeping the GATA1 levels lower, which in turn can help maintain higher apoptosis levels as a checkpoint against increased hypoxia-induced RBC production. ii) maintaining increased levels of p53 levels, which curb excessive proliferation of erythroid colony production and lower Epo sensitivity, both of which in turn prevent excessive production of RBCs under hypoxia. iii) ROS levels play a critical role in mediating the expression of GATA1 levels, and adaptive non-CMS cells maintain a good balance of ROS in cells under hypoxia. iv) ATAC-seq results confirm the changes in chromatin accessibility at the GATA1 and p53 loci. v) The effect of ARID1B on apoptosis (via GATA1) and proliferation and EPO sensitivity (through p53) was confirmed by our functional assays.

In conclusion, based on our functional results, we have determined that ARID1B is a critical regulator of erythropoiesis in the non-CMS subjects. We built a model depicting the adaptive response of ARID1B in the non-CMS subjects (Fig. 6). Under hypoxia, upregulation of ARID1B expression in the non-CMS cells regulates erythropoiesis by a) maintaining low GATA1 levels, which in turn can help maintain higher apoptosis levels as a checkpoint against increased hypoxia-induced RBC production, and b) maintaining increased levels of p53, which prevent excessive proliferation of erythroid colony production and lower Epo sensitivity. Both changes prevent excessive production of RBCs under hypoxia. Our ATAC-Seq analysis and functional experiments for GATA1 and p53 in ARID1B KD cells support the conclusion that ARID1B regulates erythropoiesis in the non-CMS high-altitude dwellers by constraining excessive RBC levels (Fig. 6). This critical role of ARID1B thereby provides an adaptive advantage to non-CMS subjects at high altitude.

## Discussion

Monge’s disease is a polygenic complex disorder^6^. Excessive erythrocytosis is strongly associated with the pathology of the disease. CMS patients at high altitude in the Andean region show severe hypoxia-mediated excessive levels of red blood cells and are therefore *maladapted*. This is especially noteworthy given that there are subjects who live side by side at the same altitude as those with CMS who do not suffer from the same extreme phenotype (non-CMS) and represent an *adapted* population in that region. Our previous whole genome analysis from both CMS and non-CMS subjects identified several candidate genes that have significant differences in haplotype polymorphism frequencies between the two groups. Our current and previous studies^1^ have helped decipher whether these genes are functionally linked to maladaptive or adaptive responses in terms of erythroid lineage activation or lack thereof under hypoxia.

In our previous work, we developed an iPSC-derived in vitro model system to investigate the role of SENP1 (a desumoylase) in the maladaptation of CMS patients. SENP1 was significantly upregulated under hypoxia in these patients, which we causally linked to the excessive erythropoiesis of CMS patients since its knockdown inhibits the exaggerated high-altitude hypoxic response^1^. In the current study, we show that the opposite effect occurs in non-CMS subjects: ARID1B is an erythropoietic regulator under hypoxia that limits the hypoxic response in non-CMS subjects. Along with the data in our previous work, we therefore provide new discoveries about the two genetic systems that control adaptation and maladaptation in the two populations at high altitudes. Indeed, in contrast to *SENP1*, *ARID1B* is significantly upregulated in non-CMS subjects, and its knockdown results in the loss of the adaptive response in constraining the excessive levels of RBCs under hypoxia.

We identified multiple roles in which ARID1B regulates various facets of erythropoiesis (Fig. 6). *First*, we discovered a distinct relationship between ARID1B and ROS in mediating erythropoietic responses under hypoxia through GATA1. *Second*, ARID1B controls proliferation (via p53) and apoptosis (through GATA1) in erythroid cells under hypoxia. *Third*, *ARID1B* controls the expression of *GATA1* and *p53* seemingly by altering their chromatin states under hypoxia.

As ARID1B is part of the SWI/SNF complex and is hypoxia dependent, it was not surprising that it influenced the cellular chromatin state to modulate the expression of specific genes in erythropoiesis^16^. This complex has been shown to alter DNA-histone contacts and nucleosome landscapes^45,46^ and can facilitate gene activation or repression by modulating chromatin accessibility at promoters and enhancers^47^. In our current study, using ATAC-Seq experiments and analyses, we discovered that *GATA1* and p53 (and their target genes) are crucial erythropoetic regulatory targets of ARID1B in the non-CMS subjects. Furthermore, the mechanistic chromatin modulating action of ARID1B is predominantly located at enhancers physically associated with transcriptionally active start sites (TSS-enhancers). A recent study by Cai et al. showed how regulation shifts from promoter-centric to enhancer-driven as cells progress developmentally from an embryonic cell stage to mature erythroid stages^48^. Enhancer-dependent regulation reflects the increased complexity of pathways and niches as encountered by adult erythroid cells^48^. Similarly, in our ATAC-Seq experiment performed at the EB stage, where the cell population is predominantly comprised of CD235a erythroid cells, we observed major changes in the enhancer regions.

The relationship between chromatin structure dynamics and hypoxia has not been clear thus far, especially regarding specific biological systems and cell types^49^. Investigations on the role of chromatin and chromatin remodeling enzymes such as SWI, particularly under hypoxia, are needed to understand the full cellular response. Although there are a few studies showing a link between HIF regulation and the SWI complex, including the fact that in vivo deletion of subparts of SWI shows lethality due to erythropoetic and vascular defects under hypoxia, the mechanistic basis for these findings is still unclear^38,50^. Our results in this study, using iPSC-derived erythroid cells from non-CMS subjects adapted to high altitude hypoxia, provide a unique platform for understanding the role of such genes. Indeed, we show here that ARID1B plays a predominant role in this adaptive response by acting as an “erythropoetic repressor”, preventing excessive erythrocytosis under hypoxia. This suppressive effect of ARID1B occurs through the dual regulation of *GATA1* and *p53* expression in erythroid cells. We believe that our findings will have implications not only for the mechanisms of hypoxia-induced excessive erythropoiesis but also for other GATA1- and p53-regulated erythropoiesis-related diseases (such as Diamond-Blackfan anemia and acute megakaryoblastic leukemia) as well as hypoxia-mediated oncogenic signaling and diseases (e.g., renal cell carcinoma, sarcomas, neuroblastoma)^14,15,51,52^.

## Supplementary information

Supplementary Information

## Data Availability

ATAC-Seq sequence files were submitted to the GEO with the GEO accession number GSE164351.
