# Supplementary material for: ARID1B, a molecular suppressor of erythropoiesis, is essential for the prevention of Monge’s disease
Source: Exp Mol Med. 2022 Jun 7;54(6):777–87. doi: 10.1038/s12276-022-00769-1 (PMC9256584; doi:10.1038/s12276-022-00769-1)
Supplement: Supplementary file 1 — Supplementary Information [file 12276_2022_769_MOESM1_ESM.pdf]

Supplementary Information and Figures:

ATAC-seq analysis explanation and significance in this study:

ARID1B is a subunit of the SWI/SNF complex, which can remodel chromatin by interfering with DNA-histone interactions in an ATP-dependent manner. As ARID1B expression is decreased in Chronic Mountain Sickness (CMS) when exposed to hypoxic conditions, we sought out to determine whether loss of ARID1B may result in dysregulation of the chromatin landscape, and if so, what type of regulatory transcription factors (TFs) may be associated with regions of chromatin with differential accessibility following ARID1B decrease. Assay for Transposase-Accessible Chromatin using sequencing (ATAC-seq) is a molecular approach to characterize the genome-wide chromatin accessibility landscape. For ATAC-seq, following purification of chromatin from cells, a hyperactive Tn5 transposase is incubated with the chromatin fraction which cuts and inserts a sequencing tag into the double-stranded DNA where it is accessible to the enzyme. These regions are amplified by qPCR and sequenced via high-throughput sequencing; sequencing reads are aligned to the genome, and peaks – regions with high concentration of reads annotated arising from localized Tn5 transposase activity (and therefore DNA accessibility) – can be computationally determined and annotated to specific genes (in promoters, enhancers, gene bodies, or intergenic regions). We performed ATAC-seq in wildtype non-CMS cells and non-CMS cells where ARID1B expression was decreased by shRNA knockdown and determined which regions of DNA had a significant change in accessibility resulting from the loss of ARID1B. We then used reference Transcriptional factor (TF) motif (i.e., the sequence of DNA preferred by a transcription factor) databases to identify the TF's whose motifs are enriched in regions of chromatin accessibility change (GimmeMotifs motif enrichment) or further to identify the TF's which have differential footprinting within regions of chromatin accessibility change (HINT-

ATAC). Finally, we use reference TF-gene target databases (e.g. ENCODE-ChEA consensus, ReMap) generated from ChIP-seq data to find the TF's whose target genes are enriched for differential accessibility within regions of chromatin accessibility change. Using these methods we identify promoter and enhancer regions that regulate the gene expression. In our current study, using this assay we have shown that ARID1B modulates the expression of GATA1 and p53 under hypoxic conditions.

Assessment of Hematopoietic potential of iPS cell lines:

We evaluated the differences in hematopoietic potential of the iPS cell lines. Supplementary fig.3 shows CD34 and CD45 +ve cells obtained per 10^7 iPS cells for each group-CMS, non-CMS and Sea level. We tested 3 cell lines (subjects) for CMS and non-CMS cells and 2 cell lines (subjects) for Sea level samples. As the results show we do not see significant differences ($P > 0.05$) between the cell lines at the hematopoietic level. For colony forming assays we do not observe significant differences ($P > 0.05$) between the colony forming potential for various colony types such as GEMM (granulocyte, erythrocyte, monocyte, megakaryocyte) at EPO level of 3 units. Supplementary Table 1 shows the data for all the colony types in CMS and non-CMS cells. Additionally, in the past, we have observed and have shown significant and drastic changes in the erythroid markers such as CD71, CD235a as well as BFU-e levels in CMS and non-CMS under hypoxia ($P<0.01$, [1]).

Supplementary Figures:

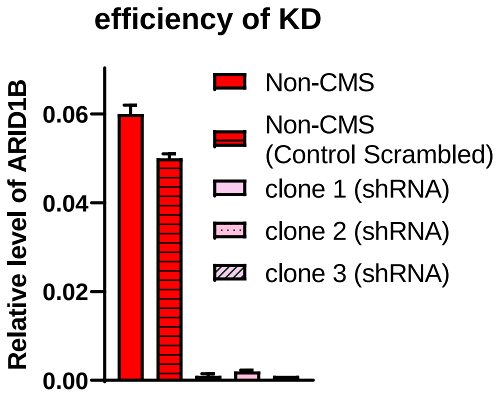

Supplementary Fig. 1: **Efficiency of KD of ARID1B by shRNA:** Fig. shows the efficiency of KD by 3 clones (1-3) with shRNA. There was a KD of >80 by these clones. N=3 subjects for each group.

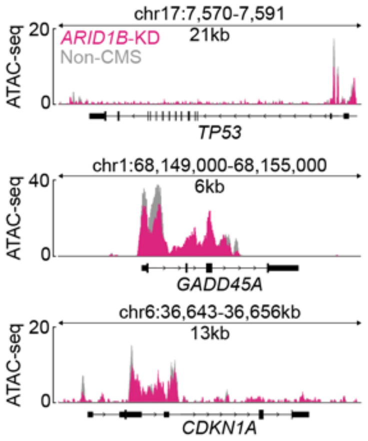

Supplementary Fig. 2: **p53 and its target genes:** ATAC-Seq read profiles around genes with decreased expression upon ARID1B KD grown under hypoxia. P53 and its target GADD45A and CDKN1A.

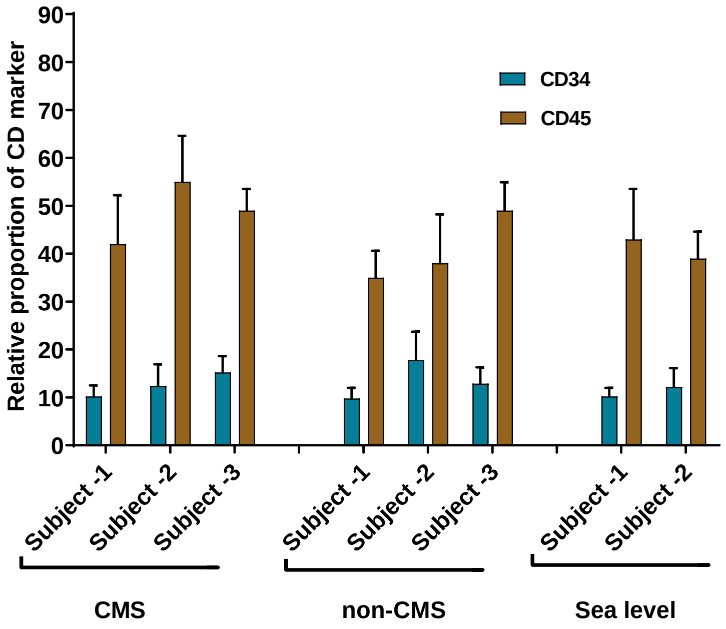

Supplementary Fig. 3: Hematopoietic potential of iPSC between CMS, non-CMS and Sea level subjects as measured by CD34 and CD45 markers.

	**CMS**	**Non-CMS**
**Colony type**	Mean±SE	Mean±SE
CFU-G	34±6.2	37±5.4
CFU-M	39±4.5	42±4.2
CFU-GM	52±4.8	50±4.5
CFU-E	37±5.2	29±2.8
CFU-GEMM	49±4.5	42±6.2

Supplementary Table 1: CFU assay results showing the colony forming potential for CMS and non-CMS cells at EPO level of 3 Units. For further information regarding erythroid lineages and BFU colonies please refer to our previous publication [1].

References:

1 Azad, P. *et al.* Senp1 drives hypoxia-induced polycythemia via GATA1 and Bcl-xL in subjects with Monge's disease. *J. Exp. Med.* **213**, 2729-2744, (2016).
